# Hollow cathode lamp based Faraday anomalous dispersion optical filter

**DOI:** 10.1038/srep29882

**Published:** 2016-07-15

**Authors:** Duo Pan, Xiaobo Xue, Haosen Shang, Bin Luo, Jingbiao Chen, Hong Guo

**Affiliations:** 1 State Key Laboratory of Advanced Optical Communication Systems and Networks, School of Electronics Engineering and Computer Science, and Center for Quantum Information Technology, Peking University, Beijing 100871, China; 2 Science and Technology on Metrology and Calibration Laboratory, Beijing Institute of Radio Metrology and Measurement, Beijing 100876, China; 3 State Key Laboratory of Information Photonics and Optical Communications, Beijing University of Posts and Telecommunications, Beijing 100876, China

## Abstract

The Faraday anomalous dispersion optical filter (FADOF), which has acquired wide applications, is mainly limited to some gaseous elements and low melting-point metals before, for the restriction of the attainable atomic density. In conventional FADOF systems a high atomic density is usually achieved by thermal equilibrium at the saturated vapor pressure, hence for elements with high melting-points a high temperature is required. To avoid this restriction, we propose a scheme of FADOF based on the hollow cathode lamp (HCL), instead of atomic vapor cells. Experimental results in strontium atoms verified this scheme, where a transmission peak corresponding to the ^88^Sr (5*s*^2^)^1^*S*_0_ − (5*s*5*p*)^1^*P*_1_ transition (461 nm) is obtained, with a maximum transmittance of 62.5% and a bandwith of 1.19 GHz. The dependence of transmission on magnetic field and HCL discharge current is also studied. Since the state-of-art commercial HCLs cover about 70 elements, this scheme can greatly expand the applications of FADOFs, and the abundant atomic transitions they provide bring the HCL based FADOFs potential applications for frequency stabilization.

Faraday anomalous dispersion optical filter (FADOF)^1^ has been widely investigated in laser stabilization and optical communication systems because of their frequency selectivity and ease of fabrication. In particular, the advantages of ultra-narrow bandwidth and high accuracy makes it an excellent candidate for laser stabilization. The application of FADOFs in laser stabilization was first realized in 1969^2^. In recent years, it has been demonstrated that the FADOF can be used in all-optical frequency locking^3^ and the building of diode lasers immune to current and temperature fluctuations^4^,^5^. Moveover, the FADOF has been directly built into an active optical clock^6^. Besides, with the characteristics of ultranarrow bandwidth^7^, high transmission, and high noise rejection^8^,^9^, the FADOFs can also be used as frequency selective components in free-space optical communication^10^ and lidar remote sensing systems^11^,^12^,^13^,^14^,^15^.

Low melting-point metals are usually used in most FADOF designs, such as mercury (254 nm)^16^, potassium (766 nm)^17^, sodium (589 nm)^18^, rubidium (780 nm^19^ and 795 nm^20^), cesium (459 nm^21^ and 894 nm^22^), and the ESFADOF of rubidium (776 nm^23^), etc. For elements with high melting-points, such as alkaline earth metals, it is much difficult to realize FADOFs due to the toughness of preparing atomic vapor with high density. In conventional method, the atomic density is determined by thermal equilibium, therefore the samples of atomic filters have to be heated to high temperatures to get an atomic density high enough to guarantee the transmittance^24^,^25^. So far, only FADOFs of calcium have been reported with complex experimental devices, such as an atomic beam^26^ and a stainless steel cell of which the sapphire viewports mounted on the ends^27^.

In order to get free from the heating problem and extend elements variety of FADOF, it is necessary to achieve high density vapor at lower temperature. Therefore it is imperative to introduce an external mechanism that provides energy to the system and break the thermal equilibium. Hollow cathode lamps (HCLs) are such products with fairly mature techniques to produce high-density atomic vapor by electric excitation. The state-of-the-art commercial HCLs cover about 70 elements (Heraeus Noblelight Hollow Cathode Lamps) and can excite large amounts of levels of neutral atoms, thus providing abundant transitions. Experimentally, the saturated absorption spectroscopy and polarization spectroscopy of several elements for frequency stabilization have been observed in the HCL, such as 423 nm and 657 nm in calcium^28^,^29^, 461 nm in strontium^30^,^31^, 399 nm in ytterbium^32^, and 377 nm in thallium^33^. Based on this, we for the first time demonstrate a HCL-FADOF working on the ^88^Sr (5*s*^2^)^1^*S*_0_ − (5*s*5*p*)^1^*P*_1_ transition (461 nm), and a preliminary experiment result with low transmittance of 28.8% was presented at IFCS 2015^34^.

In this paper, based on our preliminary experiment, a detailed study of HCL-FADOF works on the ^88^Sr (5*s*^2^)^1^*S*_0_ − (5*s*5*p*)^1^*P*_1_ transition (461 nm) is introduced. The dependence of transmission on magnetic field and HCL discharge current is carefully studied, and the transmittance has been increased to 62.5%. Some problems and the future improving directions of the HCL-FADOF scheme are also discussed. The realization of this HCL-FADOF can greatly expand the applications of FADOF, which was mainly restricted to low melting-point metal and gaseous elements before, to about 70 species of refractory elements with variety of optical transitions.

## Methods

### From heating atomic cell to hollow cathode lamp

In traditional FADOFs, the high atomic density is maintained by the saturated vapor pressure at thermal equilibrium, which needs a high temperature. Taking strontium atoms as an example, the saturated vapor pressure of strontium is given as below^35^:


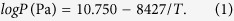


When combined with the ideal gas equation, the atomic density at different temperatures is given by:


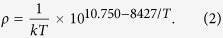


On the other hand, the atomic density can also be represented by the optical density (OD) with the definition OD = −In(*P*/*P*_0_), where *P*_0_ and *P* are probing laser powers before and after the absorption of atomic source^29^,^30^. Then the effective atomic density (those having Doppler-shifted resonant frequencies detuned from center frequency within the FWHM of the lifetime broadening) can be calculated by *P*/*P*_0_ = *e*^−*σρl*^. Combining the two expressions and considering the Doppler broadening, to get an OD of 2.6, which we obtained in the HCL experimentally, the atomic density should be as high as 2.1 × 10^16^/m^3^, hence a temperature over 300 °C is required. At this temperature, steel or sapphire has to be used instead of normal glass for atomic cells^26^, and optical coating will be much more difficult. Instead, with a discharge current of 16 mA, the same OD can be provided using HCL at room temperature. This method can greatly simplify the design of FADOF systems, and thus provide the practical method to build FADOFs of elements with high melting points at room temperature without heating.

### Experimental setup

**Figure 1:**
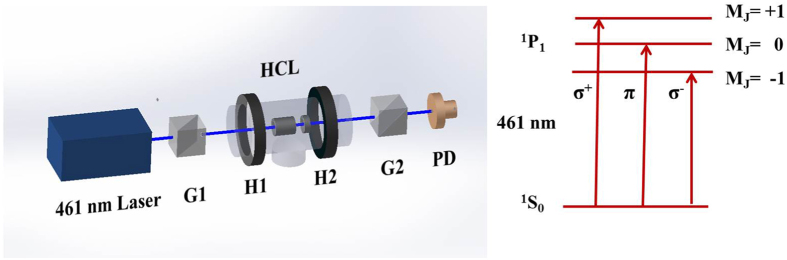
Experimental setup of the FADOF utilizing Sr hollow cathode lamp and the related energy levels. 461 nm laser: external cavity diode laser that provides the probe beam. HCL: The Sr hollow cathode lamp (HCL). G1 and G2: a pair of Glan-Taylor prisms whose polarization directions are orthogonal. H1 and H2: a pair of permanent magnets. PD: photodiode.

The experimental setup is shown in Fig. 1. The core configuration is a FADOF using a ^88^Sr HCL, with H1 and H2 being a pair of permanent magnets to generate a magnetic field up to 1748 G in the HCL center. G1 and G2 are a pair of orthogonal Glan-Taylor prisms with extinction ratio of 1 × 10^−5^. After setting in the HCL, the extinction ratio drops to 2 × 10^−2^ due to the introduced birefringence of HCL windows. A 461 nm external cavity diode laser provides the probing beam, and the transmission spectrum is detected by a photodiode (PD).

The Sr HCL (HAMAMATSU L2783), which contains inside a ring-shaped anode and a cylindrical cathode with a length of 20 mm and a bore diameter of 3 mm, is used as the atomic source, and it can easily adjust vapor pressure by regulating the discharge current. The spatial distribution of the OD in the HCL is not uniform, which is larger near the wall of the cathode^30^,^31^. In our experiment, we choose the optimal place with an OD of 2.6 at the 16 mA discharge current, which is measured under the weak light condition, when the probe beam has a waist of 0.23 mm, and the power is 50 μW. So that the intensity of probe beam is about 31 mW/cm^2^, which is below the saturation intensity of 42.75 mW/cm^2^ 36.

## Experimental Results

In our experiment, the transmittance is defined through laser powers i.e., the ratio between the transmitted and incident laser powers. When G1 is set perpendicular to G2 with the laser frequency on resonance and the magnetic field acting on the lamp, the transmitted laser power is measured by the PD to be *P*_1_. When G1 is parallel to G2 with the laser frequency far detuned and no magnetic field acting on the lamp, the transmitted laser power is measured by the PD, and then converted to the laser power on resonance, as *P*_2_, for the laser power changed linearly with the frequency in the range of measurement. The fluorescence of lamp *P*′ detected by the PD at a distance of 5.8 cm is 1.3 μW, 2.4 μW, 3.5 μW, 7.3 μW 8.7 μW and 10.0 μW under the discharge current of 7 mA, 10 mA, 13 mA, 16 mA, 19 mA and 22 mA, respectively. We define the transmittance of FADOF to be *T* = (*P*_1_ − *P*′)/(*P*_2_ − *P*′), so that the influence of the system’s optical loss has been subtracted out, for it performs equally on numerator and denominator.

**Figure 2:**
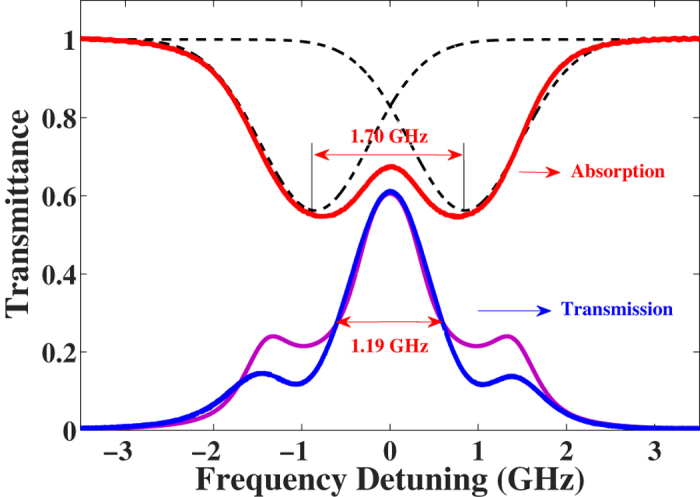
The measured absorption spectrum (red, solid), fitted absorption peaks (black, dashed), measured transmission spectrum (blue, solid), and fitted transmission spectrum (purple, solid) of the HCL-FADOF. The transmittance is 62.5% with the measured magnetic field and the discharge current being 1748 G and 16 mA, respectively. The actual magnetic field is about 607 G according to the simulation, which is less than measured because of the magnetic shielding effect in the lamp. Difference between the measured and simulated transmission spectrums results from the spacial inhomogeneity of the magnetic field and the atomic density in the HCL.

At the magnetic field of 1748 G and the HCL discharge current of 16 mA, a transmission peak with a maximum transmittance of 62.5% is obtained and the bandwidth of the filter is 1.19 GHz. The transmission spectra is shown in Fig. 2 (blue solid line). The uncertainty of the measured transmittance is 2.1%, including 2.1% from the extinction ratio of the polarizers, 0.1% from the measurement of laser power, 0.02% from the fluctuations of the magnetic field, and 0.03% from the fluctuations of the discharge current.

**Figure 3:**
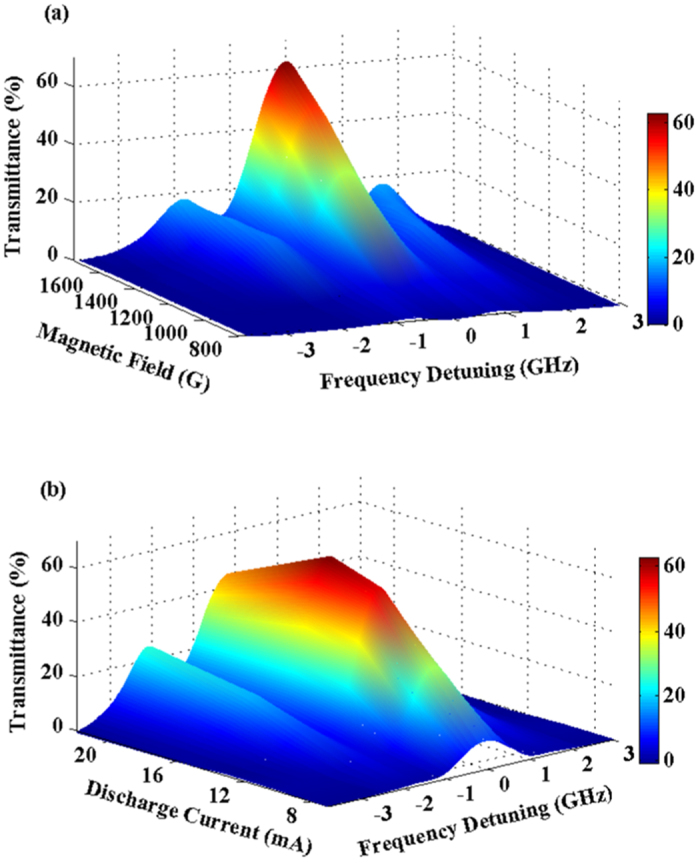
(**a**) Transmission of Sr HCL-FADOF at the fixed discharge current of 16 mA with different magnetic fields. (**b**) Transmission spectrums at the fixed magnetic field of 1748 G with different discharge currents. In both figures the magnetic fields are measured in absence of the HCL. In this paper, the measurement uncertainties of the magnetic field and the discharge current are 1 G and 1 mA, respectively.

Transmission spectrums of the Sr HCL-FADOF at different magnetic fields and lamp discharge currents are measured as in Fig. 3. Both the transmittance and the transmission bandwidth increases with the magnetic field, as shown in Fig. 3(a), and with increase of the discharge current, the transmittance increases first and then decreases, while the transmission bandwidth increases with the discharge current, as Fig. 3(b) shows.

**Figure 4:**
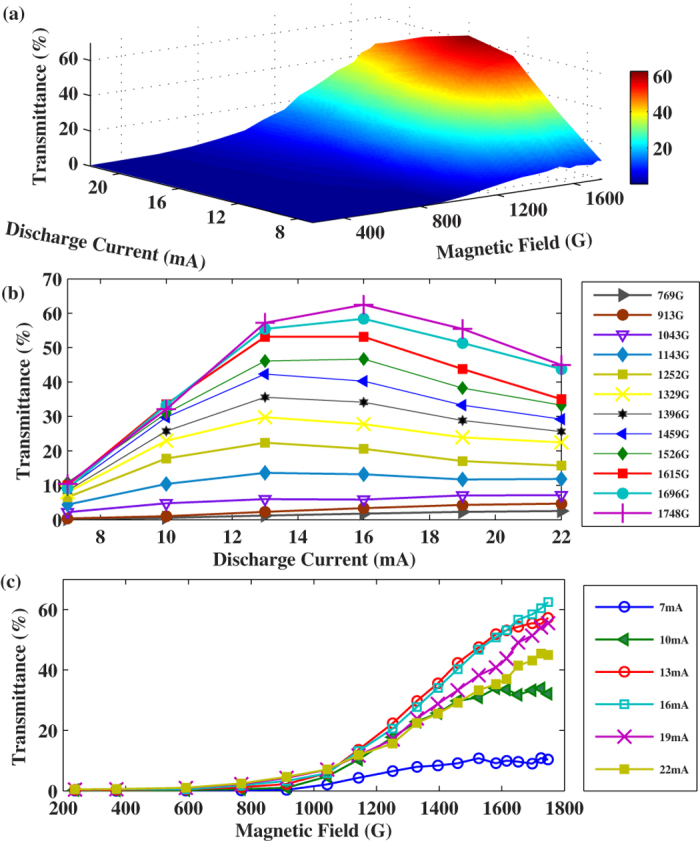
(**a**) the peak transmittance of the HCL-FADOF for various magnetic fields and discharge currents. (**b**) The peak transmittance of the Sr HCL-FADOF as a function of the HCL discharge current at different magnetic fields. (**c**) The transmittance of the Sr HCL-FADOF as a function of the magnetic field at different HCL discharge currents.

The peak transmittance of the FADOF at different discharge currents and magnetic fields is illustrated in Fig. 4. A stereogram is shown in Fig. 4(a), where the maximum transmittance (as shown in Fig. 2) occurs at the available magnetic field of 1748 G and the HCL discharge current of 16 mA. It is obvious that the transmittance can be further improved by increasing the magnetic field. Under different magnetic fields, the transmittance changes as a function of the HCL discharge current, as shown in Fig. 4(b). When the magnetic field is below 1043 G (corresponding to the figure), the transmittance slowly increases with the discharge current. When the magnetic field is above 1143 G, atomic density is the main limitation of the transmittance when the current is relatively low, the rotary power increases with atomic density and the absorption at line center remains negligible, thus the central transmittance rapidly increases with the discharge current. However, with the discharge current continue to increase, both larger atomic density and broader Doppler linewidth lead to more absorption of probe laser in the center, and finally the transmittance tapers off with the increasing discharge current. The dependence of the transmittance on the magnetic field under different discharge currents is shown in Fig. 4(c). When the HCL discharge current is below 13 mA, the transmittance of the FADOF increases with the magnetic field, and gradually converges. When the HCL discharge current is above 16 mA, the transmittance increases with the magnetic field all along in our experiment range.

## Conclusion and Discussions

In this letter, we propose a method to realize FADOFs of high melting-point elements at room temperature, with using of a HCL to maintain the high atomic density. Since the state-of-art HCLs cover about 70 elements, this method can greatly expand the application of FADOFs, which were previously restricted to low melting point metal such as mercury atoms and alkali atoms, and some gaseous elements before. Experimental results in strontium element show that a transmission peak corresponding to the ^88^Sr (5*s*^2^)^1^*S*_0_ − (5*s*5*p*)^1^*P*_1_ transition (461 nm) is obtained, with a maximum transmittance of 62.5% and a bandwith of 1.19 GHz. The dependence of transmission on magnetic field and HCL discharge current is also studied. In addition, there are still several problems worth consideration to further develop the HCL-FADOFs, as listed below.

### Fluorescence background

The HCL has a considerable fluorescence background when electric excited. In our experiment, it is detected by the photodiode at a distance of 5.8 cm to be 1.3 μW, 2.4 μW, 3.5 μW, 7.3 μW 8.7 μW and 10.0 μW under the discharge current of 7 mA, 10 mA, 13 mA, 16 mA, 19 mA and 22 mA, respectively. For this reason, the HCL-FADOF may not be suitable for the filtering of weak light, but the abundant atomic transitions they provide bring them potential applications for frequency stabilization.

### Magnetic shielding effect

It should be mentioned that the measured magnetic field strength in the experiment is not accurate. We build a stationary frame to place the HCL, and the magnetic field strength is measured in the position corresponding to the HCL center, but without the HCL. However, since the cathode cup of the lamp is made of iron, and the strontium cathode is made of Sr, Ni and Sn, which will produce a magnetic shielding effect in the lamp, the actual magnetic field in the lamp center is much smaller than 1748 G. We cannot tell the exact magnetic field strength without the magnetic conductivity of the alloy, but to give an estimated value through the Zeeman splitting of transmission spectrum. The Zeeman splitting is measured to be 1.70 GHz, corresponding to a magnetic field of 607 G. A theoretical transmission spectrum under this magnetic field is shown as the purple solid line in Fig. 2, which agrees well with the experimental data except for the height of the shoulders, due to the inhomogeneity of magnetic field and atomic density in the HCL. So we conclude that the magnetic shielding coefficient is about 2.9. Because of this effect, a larger magnetic field must be external applied to the HCL-FADOF than it is actually required. HCLs with cathode consist of nonmagnetic material may be produced to solve this problem.

### Optical loss

The optical loss ascribed to the end windows of the lamp, which is measured to be 33% in the experiment, impairs the transmitted power of FADOF. This problem can be solved by improving the window flatness and optical coating of the end windows.

### Potential application in optical clocks

The HCL-FADOF also have potential applications in optical clocks. When combined with the all-optical locking technique^3^,^4^,^5^, the FADOF can be used as an absolute frequency selector, with the transmitted light providing feedback to the laser diode instead of optical gratings. So we can realize a compact frequency-fixed Faraday laser system, in which the laser works directly at the atomic resonance line when turned on. The frequency of this laser will be immune to the fluctuations of injection current and laser diode temperature. With the abundant atomic transitions, the Faraday laser system can be used to improve the stability of optical clocks. Taking Sr optical clock for example, where 461 nm external cavity diode lasers (ECDLs) are commonly used as cooling and probing beams. These lasers are sensitive to the outside vibrations, and must be set to exact temperature and current values to keep an accurate output frequency. Hence the Faraday laser system^3^,^4^,^5^ working at ^88^Sr (5*s*^2^)^1^*S*_0_ − (5*s*5*p*)^1^*P*_1_ resonance line (461 nm) is a better alternative, which can be further stabilized to a saturated absorption spectroscopy or polarization spectroscopy, thus providing a long-term stability.

## Additional Information

**How to cite this article**: Pan, D. *et al*. Hollow cathode lamp based Faraday anomalous dispersion optical filter. *Sci. Rep.* **6**, 29882; doi: 10.1038/srep29882 (2016).
